# Problem Management Plus (PM+) in the treatment of common mental disorders in women affected by gender-based violence and urban adversity in Kenya; study protocol for a randomized controlled trial

**DOI:** 10.1186/s13033-016-0075-5

**Published:** 2016-05-31

**Authors:** Marit Sijbrandij, Richard A. Bryant, Alison Schafer, Katie S. Dawson, Dorothy Anjuri, Lincoln Ndogoni, Jeannette Ulate, Syed Usman Hamdani, Mark van Ommeren

**Affiliations:** Department of Clinical, Neuro- and Developmental Psychology, VU University Amsterdam and EMGO Institute for Health and Care Research, Van der Boechorststraat 1, 1081 BT Amsterdam, The Netherlands; School of Psychology, University of New South Wales, Sydney, Australia; World Vision International and World Vision Australia, Burwood East, Australia; World Vision Kenya, Nairobi, Kenya; World Vision Canada, Mississauga, Canada; University of Liverpool, Liverpool, UK; Human Development Research Foundation, Islamabad, Pakistan; Department of Mental Health and Substance Abuse, World Health Organization (WHO), Geneva, Switzerland

**Keywords:** Low- and middle income countries, Trauma, Gender-based violence, Intimate partner violence, Cognitive behavioural therapy, Task-shifting, Non-specialist counsellors, Common mental disorders, Depression, Anxiety, Posttraumatic stress disorder

## Abstract

**Background:**

Women affected by adversity, including gender-based violence, are at increased risk for developing common mental disorders such as depression, anxiety and posttraumatic stress disorder (PTSD). The World Health Organization (WHO) has developed Problem Management Plus (PM+), a 5-session, individual psychological intervention program, that can be delivered by non-specialist counsellors that addresses common mental disorders in people affected by adversity. The objectives of this study are to evaluate effectiveness of PM+ among women who have been affected by adversity, including gender-based violence, and to perform a process evaluation.

**Methods:**

Informed by community consultations, the PM+ manual has been translated and adapted to the local context. A randomized controlled trial will be carried out in the catchment areas of three local health care facilities in Dagoretti Sub County, Nairobi. After informed consent, females with high psychological distress (General Health Questionnaire-12 (score >2) and functional impairment (WHO Disability Assessment Schedule 2.0 score >16) will be randomised to PM+ (n = 247) or enhanced treatment as usual (n = 247). Post-treatment and 3-months post-treatment follow-up assessments include psychological distress, functional disability, PTSD symptoms, perceived problems for which the person seeks help, health care use and health costs. For evaluating the process of implementing PM+ within local communities in Nairobi 20 key informant interviews will be carried out in participants, PM+ providers, decision makers, clinical staff.

**Discussion:**

If PM+ is proven effective, it will be rolled out to other low and middle income areas and other populations for further adaptation and testing.

*Trial registration* Australian New Zealand Clinical Trials Registry, ACTRN12616000032459. Registered prospectively on January 18, 2016

## Background

Gender-based violence (GBV) such as intimate partner violence or sexual violence by someone else is an increasing global topic of concern. Worldwide, the estimated lifetime prevalence of GBV is at least 30 % [1–3] Women living in urban poverty—including slums—in low and middle income countries (LMICs) are especially vulnerable for violence and common mental disorders, since poverty poses women at risk for experiencing gender-based violence. In Kenya, 47 % of women reported having experienced physical and/or sexual violence at some point in their lives.

Women exposed to GBV are at higher risk for developing common mental disorders such as depression, anxiety disorders, and posttraumatic stress disorder (PTSD) and make more use of health services than non-abused women [2, 4, 5]. Common mental disorders are among the largest contributors to disability and functional impairment (see Patel et al. [6]). Additional consequences of GBV involve reduced social and economic development prospects for women because of unwanted pregnancy, lack of resources, limited opportunities, concurrent physical health problems, low education, and fewer social supports [7, 8]. In most communities, women affected by GBV tend to receive no or minimal mental health support [1].

Effective interventions to treat common mental disorders are available (e.g., cognitive behavioural therapy; CBT [9, 10]). However, these interventions typically require expert mental health professionals providing treatments that are usually lengthy and costly to the health service. LMICs lack sufficient numbers of specialized mental health care professionals to deliver such interventions and are unlikely to afford costs associated with scaling up intensive treatment programs in an effective but safe manner. For practically and sustainably scaling-up evidence-based mental health care interventions in LMICs, interventions should be short and relatively easy to administer, so they can be carried out by non-specialist helpers in the community, such as community health workers (cf. [11]). In addition, interventions should not target one outcome (e.g. PTSD) but address a broader range of outcomes, including general functioning and common mental and psychosocial health problems.

World Health Organization (WHO) has developed a low-intensity 5-sessions program termed Problem Management Plus (PM+) which may be delivered by trained non-specialist helpers [12]. PM+ aims to reduce symptoms of depression, anxiety, PTSD, stress in the wake of adversity and trauma and to improve functioning. It comprises evidence-based techniques: of (a) problem solving, (b) stress management, (c) behavioral activation, and (d) accessing social support.

The impetus for developing the PM+ manual came from request to the Department of Mental Health and Substance Abuse at the WHO in Geneva to develop interventions for survivors of GBV that may be delivered in such manner that they are feasible in Sub-Saharan Africa, where (a) health systems typically do not have the resources to make available proven psychological interventions on a routine basis (e.g. exposure therapy, cognitive processing therapy, Eye Movement Desensitization and Reprocessing), and (b) where social stigma against GBV can be so high that attending specific mental health care for GBV can put women at social risk (e.g., further violence, abandonment by the community). PM+ addresses these two requirements by involving relatively simple, non-intrusive techniques that do not necessarily focus on the trauma of GBV. It can thus be offered outside of the context of vertical GBV-services by briefly trained but well-supervised helpers to most women impaired by distress, irrespective of the causes of the distress. Additionally, this study aims to test the capacity of PM+ to reduce mental disorders in women affected by GBV by screening for psychological distress in women in adversity-affected community settings; because this way of case identification potentially overcomes the barrier of GBV-survivors to seek mental health assistance and reduces the risks of stigmatizing women who have suffered GBV through services that are only for GBV survivors.

## Methods

### Aim and design

**Fig. 1:**
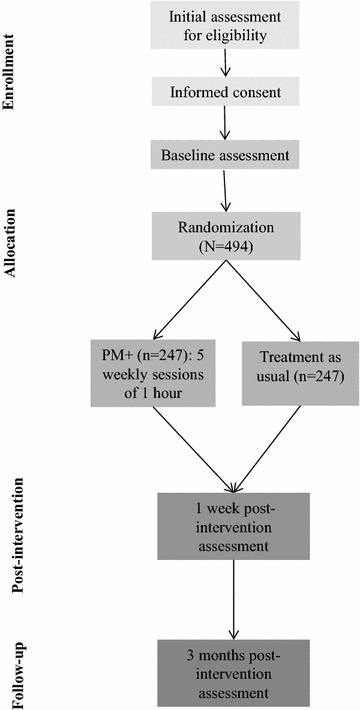
Flow diagram

This study aims to evaluate the effectiveness of the locally adapted version of PM+ in women affected by adversity and GBV in urban slum areas in Nairobi, Kenya in reducing symptoms of common mental disorders. We will conduct a randomized controlled trial (RCT) comparing PM+ to enhanced treatment as usual (ETAU) in 494 study participants. See Fig. 1 for an overview of the design. In addition, we will perform a process evaluation to examine barriers and facilitators when implementing the RCT and the interventions in the chosen settings.

### Setting

The study will be carried out in three local health care facilities that are part of the primary healthcare system of the Dagoretti Sub County Nairobi in Kenya. Kenya is a LMIC; despite a growing middle class, almost half (45.9 %) of the population lives below the national poverty line. The study is overseen by staff of World Vision Kenya and their Riruta Area Development Program, which is a geographic area where World Vision implements long-term community development programs in collaboration with primary health care centres.

### Participants

Participant inclusion criteria are: (a) adult (18 years or older) females; (b) score above 2 on a screening questionnaire for psychological distress (General Health Questionnaire-12; GHQ-12; and (c) score above 16 on a screening questionnaire for functional impairments (WHO Disability Assessment Schedule 2.0; WHODAS; [13]). These instruments are described below. Exclusion criteria are: (a) male gender; (b) acute medical conditions; (c) risk of suicide; or (d) severe mental disorders or cognitive impairment (e.g., severe intellectual disability, dementia, psychosis).

### Informed consent

Informed consent entails a two-step procedure: (1) Informed consent for screening and (2) Informed consent for taking part in the PM+ trial. The latter is only required for participants meeting inclusion criteria. For each step respondents who decide to participate will be asked to complete a written consent form. Each person will be given at least 24 h to think whether she wants to take part in the PM+ trial. For participants who are illiterate, witnessed oral consent and a thumb print in lieu of a signature will be asked, in line with recommendations from WHO [14]. The witness will not be a member of the research team.

### Procedure

Participants will be included through community screening at the homes of the participants by independent assessors. Assessors start sampling at a central point in the community and randomly select a direction in which to screen, selecting households using a population-based interval approach. In this approach, members from a larger population are selected according to a random starting point and a fixed, periodic interval (called the sampling interval), which is calculated by dividing the population size by the desired sample size. Thus, the sampling interval will vary depending on the population size of each of the three areas involved in the research.

After selecting the households, independent assessors will identify and attempt to meet with the head of each household. The assessors explain the purpose of the survey and ask if they may interview a random adult woman aged 18 years or older from the household. If a household does not have a woman of 18 years or older, or the woman declines to be screened for the research, the enumerator moves on to the next household based on the sampling intervals.

The independent assessors will refer individuals meeting any of the exclusion criteria to specialist support in the county according to their need. All interviews are conducted face-to-face in a private space (e.g. in people’s homes or in a quiet area near their homes). If participants are not selected because they score below the cut-offs for either the GHQ-12 or the WHODAS, they will be provided feedback on their test outcomes and reasons why they are not eligible for the study will be explained to them. Next, the pre-assessment is completed by administering the PTSD Checklist for DSM-5 (PCL-5), Life Events Checklist (LEC), selected questions of the WHO violence against women instrument (WHO-VAW), the psychological outcome profiles instrument (PSYCHLOPS) and items derived from the Service Receipt Inventory (SRI).

The post-intervention assessment (WHODAS, GHQ-12, PCL-5, PSYCHLOPS) is scheduled 7 weeks after the pre-intervention assessment (i.e., 1 week after the 5th PM+ session), and the follow-up assessment (WHODAS, GHQ-12, PCL-5, LEC, PSYCHLOPS, SRI) is scheduled at 13 weeks after the post-intervention assessment (i.e. 20 weeks after inclusion, in line with the timing of the follow-up assessment for the PM+ participants). The participants who receive PM+ will also be administered the PSYCHLOPS by the non-specialist helpers at the beginning of each PM+ session. Table 1 presents an overview of measures that are administered at each of the assessments.

**Table 1:** Overview of measures administered at the assessments

Concept	Pre-assessment measures	Post-treatment assessment measures	3-months post-treatment follow-up assessment measures
1. Psychological distress	GHQ-12 (*screener and primary outcome*)	GHQ-12	GHQ-12
2. Functioning	WHODAS (*screener*)	WHODAS	WHODAS
3. PTSD symptoms	PCL-5	PCL-5	PCL-5
4. Perceived problems	PSYCHLOPS	PSYCHLOPS	PSYCHLOPS
5. Adverse life events	LEC		LEC
6. Violence against women	WHO-VAQ		
7. Costs of care	SRI items		SRI items

All instruments will be delivered in interview format as many participants are expected to be illiterate. Female independent assessors, most holding a tertiary-level degree in psychology or a related discipline, and fluent in Kiswahili and English will carry out the screening and pre and post assessments. Assessors will have received a three-day training covering administering the instruments, common mental disorders, general interview techniques, and ethical research conduct. An additional one-day training in psychological first aid and how to support women experiencing emotional distress will also be provided for assessors. Ongoing monitoring of assessors’ competency will be conducted through regular supervision by the trial manager. The assessors will be blind to the allocation status of the participants.

### Sample size

A total number of 494 participants will to be included. Since we are not aware of intervention studies that have been carried out in this population, and we expect the population to be heterogeneous with respect to the types of common mental disorders, we aimed for a small to medium effect size of *d* = 0.04 in GHQ-12 symptom score in the PM+ group as compared to the control group at 3 months after the conclusion of the study. These estimates are in line with the observed effectiveness of an intervention led by lay health counsellors for depressive and anxiety disorders in a study in primary care in India [15]. Power calculations suggest a minimum sample size of 133 participants per group (power = 0.95, alpha = 0.05, two-sided). Taking into account 30 % attrition at follow-up, at least 346 participants (173 per group) need to be included. Further, since we estimate that 70 % of women in this distressed sample would have a history of GBV, we intend to include at least 494 women in the study.

### Randomization

Randomization will occur following pre-assessment. This will be conducted by an independent research assistant located off-site (University of New South Wales) and not involved in any other aspects of the study. Randomization will be performed using computerized software on a 1:1 basis.

### The Problem Management Plus (PM+) program

The WHO PM+ program involves a set of a brief psychological strategies that seek to ameliorate symptoms of common mental health problems (e.g. depression, anxiety, stress). The intervention protocol was written for WHO by a consultant of the University of New South Wales, Australia [12, 16].

The PM+ intervention protocol has been translated into Kiswahili and adapted to the local sociocultural context of the lives of women in poor neighborhoods of Nairobi. The translation and adaptation have been reviewed in two workshops: one with experts on the intervention and the other with community representatives including local leaders, local health care provides, community health workers (CHWs) and Kenyan translators [17].

PM+ integrates problem-solving and behavioral treatment techniques that demonstrate amenability to low-intensity delivery and are evidence-based [18–21]. PM+ is delivered over 5 weekly sessions of 90 min duration. Clients are systematically taught four strategies: (1) stress management; (2) problem solving; (3) behavioral activation and (4) skills to strengthen social support. The PM+ program is being made available in different formats. In the current RCT, the PM+ individual version will be tested.

PM+ providers will be female CHWs, who will be selected based on individual interviews. PM+ providers will meet the following criteria: (1) High school diploma (form 4 level of education or above); (2) Reasonable proficiency in both Kiswahili and English language; (3) 1-year training post-secondary school as CHWs or health related career; (4) 2-years and above experience in community work; and (5) Already working as a CHW within a given geographical area. PM+ providers will receive a total of 2 weeks of training. In addition, one professional Kenyan supervisor will be employed for each of the three health facility areas.

Protocol adherence will be ensured by the supervisors and weekly group supervisions of the CHWs [22]. Supervisors will receive weekly supervision and on-the-job training in supervision skills by the project intervention consultant (KSD).

To evaluate treatment fidelity, 10 % of all PM+ sessions will be attended by the supervisor, using a checklist to ensure basic elements of the PM+ intervention have been followed as required.

At the beginning of every PM+ session the PSYCHLOPS will be administered. This instrument, in contrast to above named instruments will be administered by the CHW to assess and monitor progress on problems for which the person seeks help.

### Enhanced treatment-as-usual (ETAU)

Treatment-as-usual in primary healthcare centers in Nairobi, Kenya to individuals with common mental disorders usually consists of no or placebo treatment. For this study, treatment as usual will be substantially enhanced. Participants in the ETAU group will be referred to their primary care clinicians (usually nurses) for follow-up. These primary care nurses will have received a 3-day training; 1 day on basic psychological first aid communication skills and building on this with training from the International Federation of Red Cross non-specific lay-counselling resource [23]. This resource focuses on ethical considerations for lay counsellors, structuring a helping conversation and referral for high care.

We will seek to keep track of the types and amount of support participants receive through the SRI. If, during this treatment or during the study’s assessments participants in ETAU arm show severe psychiatric disorders or problems (e.g., psychosis or suicidality) that require immediate specialist treatment and follow-up, they will be referred to the subcounty hospital, which has been contracted to provide gratis care to the participants referred for these problems.

### Screening measures

The WHODAS [13] is a generic assessment instrument assessing general functioning, health and disability. Simple to administer, it is applicable across all health states, including mental disorders, and across cultures. WHODAS covers six domains (cognition, mobility, self-care, getting along, life activities, and participation). It assesses difficulties people have due to their illness across these domains during the last 30 days. Difficulties are scored as none, mild, moderate, severe, or extreme. We will use the 12-item interviewer administered version. The WHODAS has been validated in Kenya.

The GHQ-12 [24, 25] assesses level of general psychological distress during screening. The GHQ-12 consists of 12 questions that are scored on a 4-point scale ranging from 0 to 3. The total GHQ-12 score is obtained by summing up the scores of the individual items and ranges between 0 and 36 with higher scores representing higher levels of distress. When used as a screening tool, the GHQ-12 is usually scored bi-modally (i.e.-0-0-1-1), and ranges between 0 and 12. In a previous study in Kenya, a cut-off of higher than 2 has been reported to indicate clinical levels of distress [26].

Data on socio-demographic information (sex, age, education, marital status and work status) will be collected through questions A1-A5 of the WHODAS.

### Primary outcomes

Primary outcomes are the level of psychological distress as measured with the GHQ-12 at 3 months follow-up (i.e., 3 months after the 5th PM+ session) and general functioning as measured with the WHODAS.

### Secondary outcomes

Posttraumatic stress disorder (PTSD) symptoms will be measured using the PCL-5 [27], which is a 20-item checklist corresponding with the 20 DSM-5 PTSD symptoms. Items are rated on a 0–4 scale and add up to a total severity score of 80. The PCL-5 will be adapted to ask for symptoms in the last week (rather than month) to enhance sensitivity to change.

PSYCHLOPS [28] will be administered at all assessments and at the beginning of each PM+ session, and assesses progress on problems for which the person seeks help. It consists of four questions that encompass three domains: problems (2 questions), functioning (1 question) and wellbeing (1 question). Participants are asked to give free text responses to the problem and function domains. Responses are scored on an ordinal six-point scale producing a maximum score of 20 (5 points per question). The PSYCHLOPs version administered at posttreatment and follow-up also includes an overall valuation question (determining self-rated outcome ranging from “much better” to “much worse”). PSYCHLOPS has been validated in primary care populations across several countries.

### Other measures

Previous stressor exposure will be assessed using the LEC [29]. This is a widely used list of 17 experienced or witnessed events, such as rape, serious injury, combat exposure, or the sudden death of a loved one. A Kiswahili version of this list is available [30]. At post-test the question phrasing will be adapted to capture life events that have occurred since commencing in the trial.

Five key questions of the Swahili version of the WHO-VAW as developed for use in the WHO Multi-Country Study on Women’s Health and Domestic Violence [31] are administered.

Indicators of economic impact will be assessed using the WHODAS question on days out of role and by selected SRI [32] questions on health care service use.

### Process evaluation

The feasibility, and difficulties and successes in carrying out research and intervention activities will be explored through semi-structured interviews with 20 key informants, including 5 CHWs. The burden of completing the assessments and PM+ on the time and effort of participants, satisfaction with the intervention, and barriers and facilitators to adherence will be explored through semi-structured interviews with a sample of 5 participants (including participants that have dropped out). In addition, 5 decision-makers with responsibilities for developing or implementing health policy, including heads of the relevant clinics, and 5 PHC staff (clinical officers/nurses) will be interviewed to obtain their perceptions of the benefits and challenges of integrating PM+ into the CHWs routine service provision.

### Analysis

To determine comparability between the conditions at baseline, multiple planned comparisons will be conducted for continuous variables and Chi squared tests for categorical ones.

Hierarchical linear modeling (HLM) analysis will be carried out to assess differential change over time in GHQ, WHODAS, and PSYCHLOPS scores between groups. For each outcome, the effects of time of measurement, group, and the group-by-time interaction will be analyzed. HLM presumes intent-to-treat analyses as HLM allows the number of observations to vary between participants and effectively handles missing data. Time (linear and quadratic), treatment condition, and their interaction will be included in the models. Fixed effects parameters will be tested at 95 %CI. The Level 1 model will represent within-patient change over time, and the Level 2 model will predict variation in within-patient change over time and encompass between-patient variables. Outcome analyses will be reported primarily in women reporting GBV, and secondarily for women affected by all forms of adversity.

Descriptive analyses will be carried out in SPSS and HLM analyses in Stata version 11.2. Across all analyses, two-tailed tests will be reported with *p* < 0.05.

### Adverse events reporting

All adverse reactions and serious adverse events (SAEs) reported spontaneously by the subject or observed by the investigators or other staff members will be recorded by the research team. A SAE is an undesirable experience occurring to a subject during the study, whether or not considered related to the research procedure. Although it is unlikely that SAEs would occur given the nature of the intervention, all adverse events and SAEs will be reported to the local independent advisory board. This board will consist of the supervisor, the study coordinator and an independent medical professional from the local health facility. The chair or a nominated person from the advisory board will review SAEs within 48 h and the advisory board will review all AEs twice a month and where necessary to determine any appropriate action in respect of ongoing trial conduct. On the informed consent form, patient information is included to inform participants that the field coordinator, or another clinician other than their therapist are available to them if they are upset by this study.

### Ethics

The project has been approved locally by the Research Ethics Committee of the Great Lakes University of Kisumu, Kenya and by the WHO Ethical Review Committee (Protocol ID: RPC656, April 25, 2014, Amendment February 16, 2015).

## Discussion

By evaluating the effectiveness of PM+ in women affected by gender-based violence in Nairobi, Kenya, we seek to address the need for access to brief, effective psychological interventions that can be administered by non-professional health workers in LMICs. A second RCT on the effectiveness of individually delivered PM+ in males and females in an humanitarian setting in Peshawar, Pakistan is underway [33]. If proven effective, PM+ may not only be used in similar areas in Kenya and Pakistan, but rolled out to other affected areas in both LMIC settings and high-resource countries for further adaptation. It may be a useful intervention for humanitarian settings (e.g., conflict settings) where mental health care for GBV-survivors is often lacking. PM+ may be tested in different formats (e.g., internet-delivered, group or young adolescent version) across a variety of populations suffering from psychological distress. The PM+ manual and accompanying training materials, if proven effective, will be published by WHO and will be made available on WHO’s website, in order to enable future scaling-up.

## Abbreviations

WHO: World Health Organization
PM+: Problem Management Plus
RCT: randomized controlled trial
GBV: gender based violence
WHODAS: WHO Disability Assessment Schedule 2.0
GHQ-12: General Health Questionnaire-12
SRI: Service Receipt Inventory
ETAU: enhanced treatment as usual
PTSD: posttraumatic stress disorder
LMIC: low and middle income country
CBT: cognitive behavioural therapy
PCL-5: PTSD checklist for DSM-5
WHO-VAW: WHO violence against women instrument
LEC: life events checklist
PSYCHLOPS: psychological outcome profiles instrument
CHW: community health worker
HLM: hierarchical linear modeling
SPSS: statistical package for the social sciences
SAE: serious adverse event

## References

[1] World Health Organization (2013). Global and regional estimates of violence against women: prevalence and health effects of intimate partner violence and non-partner sexual violence.

[2] Rees S (2011). Lifetime prevalence of gender-based violence in women and the relationship with mental disorders and psychosocial function. JAMA.

[3] Devries KM (2013). Global health. The global prevalence of intimate partner violence against women. Science.

[4] Ellsberg M (2008). Intimate partner violence and women’s physical and mental health in the WHO multi-country study on women’s health and domestic violence: an observational study. Lancet.

[5] Bonomi AE (2009). Health care utilization and costs associated with physical and nonphysical-only intimate partner violence. Health Serv Res.

[6] Patel V (2016). Addressing the burden of mental, neurological, and substance use disorders: key messages from disease control priorities, 3rd edition. Lancet.

[7] Dibaba Y, Fantahun M, Hindin MJ (2013). The association of unwanted pregnancy and social support with depressive symptoms in pregnancy: evidence from rural Southwestern Ethiopia. BMC Pregnancy Childbirth.

[8] Garcia-Moreno C (2015). Addressing violence against women: a call to action. Lancet.

[9] Cuijpers P (2013). The efficacy of psychotherapy and pharmacotherapy in treating depressive and anxiety disorders: a meta-analysis of direct comparisons. World Psychiatry.

[10] Bisson JI (2007). Psychological treatments for chronic post-traumatic stress disorder. Systematic review and meta-analysis. Br J Psychiatry.

[11] Rahman A (2008). Cognitive behaviour therapy-based intervention by community health workers for mothers with depression and their infants in rural Pakistan: a cluster-randomised controlled trial. Lancet.

[12] Dawson KS (2015). Problem Management Plus (PM +): a WHO transdiagnostic psychological intervention for common mental health problems. World Psychiatry.

[13] World Health Organization (2010). Measuring health and disability; manual for WHO disability assessment schedule WHODAS 2.0.

[14] World Health Organization. The process of obtaining informed consent. http://www.who.int/ethics/review-committee/guidelines/en/. Accessed 4 Sept 2013.

[15] Patel V (2010). Effectiveness of an intervention led by lay health counsellors for depressive and anxiety disorders in primary care in Goa, India (MANAS): a cluster randomised controlled trial. Lancet.

[16] World Health Organization (2016). Problem Management Plus (PM +): individual psychological help for adults impaired by distress in communities exposed to adversity (Generic field-trial version 1.0).

[17] World Health Organization (2016). Problem Management Plus (PM +): individual psychological help for adults impaired by distress in communities exposed to adversity (Kenyan version).

[18] Bennett-Levy J, Richards D, Farrand P (2010). Oxford guide to low intensity CBT interventions.

[19] Farchione TJ (2012). Unified protocol for transdiagnostic treatment of emotional disorders: a randomized controlled trial. Behav Ther.

[20] van’t Hof E (2011). The effectiveness of problem solving therapy in deprived South African communities: results from a pilot study. BMC Psychiatry..

[21] Cuijpers P, van Straten A, Warmerdam L (2007). Problem solving therapies for depression: a meta-analysis. Eur Psychiatry.

[22] Murray LK (2011). Building capacity in mental health interventions in low resource countries: an apprenticeship model for training local providers. Int J Ment Health Syst.

[23] Psychosocial Centre, International Federation of Red Cross and Red Crescent Societies, War Trauma Foundation, Danish Cancer Society & University of Innsbruck. Lay counselling: a trainers manual. Amsterdam: IFRC; 2013.

[24] Goldberg DP, Williams P (1988). A user’s guide to the General Health Questionnaire.

[25] Minhas FM (1996). MH Validation of General Health Questionnaire (GHQ-12) in primary care settings of Pakistan. J Coll Phys Surg Pak.

[26] Jenkins R (2013). Short structured general mental health in service training programme in Kenya improves patient health and social outcomes but not detection of mental health problems - a pragmatic cluster randomised controlled trial. Int J Ment Health Syst.

[27] Weathers FWL, Litz BT, Keane TM, Palmieri PA, Marx BP, Schnurr PP. The PTSD checklist for DSM-5 (PCL-5). 2013. Scale available at from the National Center for PTSD at http://www.ptsd.va.gov.

[28] Ashworth M, Shepherd M, Christey J, Matthews V, Wright K, Parmentier H, Robinson S, Godrey E (2013). A client-generated psychometric instrument: the development of “PSYCHLOPS”. Couns Psychother Res Link Res Pract.

[29] Gray MJ (2004). Psychometric properties of the life events checklist. Assessment.

[30] Whetten K (2011). More than the loss of a parent: potentially traumatic events among orphaned and abandoned children. J Trauma Stress.

[31] World Health Organization (2005). WHO multi-country study on women’s health and domestic violence against women.

[32] Chisholm D (2000). Client socio-demographic and service receipt inventory-European version: development of an instrument for international research. EPSILON Study 5. European psychiatric services: inputs linked to outcome domains and needs. Br J Psychiatry Suppl.

[33] Sijbrandij M (2015). Problem Management Plus (PM +) for common mental disorders in a humanitarian setting in Pakistan; study protocol for a randomised controlled trial (RCT). BMC Psychiatry.

