# Correction to: Problem Management Plus (PM+) in the treatment of common mental disorders in women affected by gender-based violence and urban adversity in Kenya; study protocol for a randomized controlled trial

**DOI:** 10.1186/s13033-018-0236-9

**Published:** 2018-10-12

**Authors:** Marit Sijbrandij, Richard A. Bryant, Alison Schafer, Katie S. Dawson, Dorothy Anjuri, Lincoln Ndogoni, Jeannette Ulate, Syed Usman Hamdani, Mark van Ommeren

**Affiliations:** 1 Department of Clinical Neuro- and Developmental Psychology, VU University Amsterdam and EMGO Institute for Health and Care Research, Van der Boechorststraat 1, 1081 BT Amsterdam, The Netherlands; 2 School of Psychology, University of New South Wales, Sydney, Australia; 3 World Vision International and World Vision Australia, Burwood East, Australia; 4 World Vision Kenya, Nairobi, Kenya; 5 World Vision Canada, Mississauga, Canada; 6 University of Liverpool, Liverpool, UK; 7 Human Development Research Foundation, Islamabad, Pakistan; 8 Department of Mental Health and Substance Abuse, World Health Organization (WHO), Geneva, Switzerland

## Correction to: Int J Ment Health Syst (2016) 10:44 10.1186/s13033-016-0075-5

In the original version of this article [1], the reference 28 was published incorrectly. The corrected reference is given below:

Ashworth M, Shepherd M, Christey J, Matthews V, Wright K, Parmentier H, Robinson S, Godfrey E. A client-centred psychometric instrument: the development of ‘PSYCHLOPS’ (‘Psychological Outcome Profiles’). *Counselling and Psychotherapy Res* 2004;4:27–33.
